# Diversity and Distribution of *Hyalomma* Ticks and Tick-Borne Pathogens in Dromedary Camels in Chad

**DOI:** 10.3390/vetsci13050443

**Published:** 2026-04-30

**Authors:** Muhammad Umair Aziz, Jacob Cassens, Jeconias Allawaï-Sanigue, Michel Lontsi-Demano, Timoléon Tchuinkam, Olivier Andre Sparagano, Jonathan D. Oliver, Patrick Butaye

**Affiliations:** 1 Department of Infectious Diseases and Public Health, Jockey Club College of Veterinary Medicine and Life Sciences, City University of Hong Kong, Kowloon, Hong Kong SAR, China; pabutaye@cityu.edu.hk; 2 Division of Environmental Health Sciences, School of Public Health, University of Minnesota, Minneapolis, MN 55455, USA; 3 Vector Borne Diseases Laboratory of the Research Unit of Biology and Applied Ecology (VBID-RUBEA), Department of Animal Biology, Faculty of Sciences, University of Dschang, Dschang P.O. Box 067, Cameroon; 4 International Institute of Tropical Agriculture (IITA), Cotonou P.O. Box 0932, Benin; 5 Angila Ruskin University, 2 Clove Crescent, London E14 2BE, UK; 6 Department of Pathobiology, Pharmacology and Zoological Medicine, Faculty of Veterinary Medicine, Ghent University, Salisburylaan 133, 9820 Merelbeke, Belgium

**Keywords:** *Hyalomma dromedarii*, *Hyalomma rufipes*, tick-borne pathogens, *Coxiella burnetii*, *Rickettsia aeschlimannii*, Chad

## Abstract

Ticks of the genus *Hyalomma* are major ectoparasites of dromedary camels and serve as important vectors for diverse tick-borne pathogens (TBPs) affecting both animals and humans. In this study, we determined the tick species diversity and distribution and estimated the prevalence of TBPs in those ticks. A total of 780 ticks collected from camels in Bol, Chad, were identified into four species: *Hyalomma dromedarii* (49.0%), *H. rufipes* (22.6%), *H. impeltatum* (19.1%), and *H. truncatum* (9.4%). Sixty ticks were selected proportionally across the four *Hyalomma* species and screened for TBPs using PCR. *Coxiella burnetii* was detected in 11.7% of the ticks, and *Rickettsia aeschlimannii* in 1.7%. Anaplasmataceae-specific *16S rRNA* primers detected *Candidatus Midichloria mitochondrii*, a tick endosymbiont, in 10% of the ticks. No protozoan pathogens (*Theileria* or *Babesia*) were detected. This study highlights the need for integrated surveillance of ticks and their associated microorganisms in Chadian camels to mitigate zoonotic and veterinary risks. Strengthening such efforts will support camel health and pastoral livelihoods in the region.

## Simple Summary

Camels are very important for people’s livelihoods in Chad, but ticks can seriously affect their health. In this study, we investigated tick species living on one-humped camels around Bol in Chad and screened them for tick-borne pathogens (TBPs) that can make both animals and people sick. We collected 780 ticks and identified four species, with *Hyalomma dromedarii* being the most common. We detected *Coxiella burnetii*, the bacterium that causes Q fever in humans, and a spotted fever group *Rickettsia* that can also cause illness in people. We did not detect any blood parasites, such as *Babesia* or *Theileria*, in these ticks. These results show that ticks on camels can carry diseases that may spread to people in camel-rearing communities. Even though the number of infected ticks was low, regular checks and tick control are important to protect both camels and the families who depend on them.

## 1. Introduction

Camels (*Camelus dromedarius*) are essential to the livelihoods of agropastoral communities in Chad, a country that hosts one of the world’s largest camel populations, estimated at approximately 9–10 million heads according to recent FAOSTAT data [1]. These resilient animals are well-suited for arid and semi-arid environments, where other livestock often face significant challenges related to limited water and forage availability [2]. Their adaptability makes them the cornerstone of food security and is crucial for transportation and trade in these regions. The economic significance of camels is underscored by their role in Chad’s livestock sector, which contributes substantially to the national economy, accounting for approximately 18% of the national GDP [3]. Maintaining camel health is therefore essential for economic stability, food security, and resilience in Sahelian production systems.

Despite their adaptation to harsh environments, camels are affected by parasitic diseases that can reduce productivity. Among these, ticks are considered one of the most important ectoparasites of camels [4,5], with *Hyalomma* ticks most frequently reported [6]. Infestations result in significant economic losses due to decreased milk and meat production, hindered growth rates, increased vulnerability to secondary infections, and damage to hides, which reduces their commercial value and suitability for trade or leather processing [7,8]. In addition to these direct effects, ticks act as vectors or carriers of a wide range of microorganisms of veterinary and public health relevance, including *Theileria* spp., *Babesia* spp., Anaplasmataceae (such as *Anaplasma* and *Ehrlichia* spp.), spotted fever group (SFG) *Rickettsia* spp., and *Coxiella burnetii* [9,10].

Despite their considerable impact, the presence and prevalence of tick-borne diseases in Chadian livestock remain largely unknown [11]. Zoonotic pathogens such as *Rickettsia africae* and *R. aeschlimannii* (spotted fever group) have been detected in ticks infesting livestock in Chad and neighbouring regions, posing significant risks to animal and human health [12,13,14]. Camels have recently been identified as potential reservoirs for several of these pathogens, including *Coxiella*, *Rickettsia*, and *Anaplasma*, highlighting the public health importance of tick infestations in camel-rearing systems [10,14,15].

The Lac region is one of Chad’s main agro-sylvo-pastoral areas. Livestock production is highly developed along the shores of Lake Chad, and transhumant pastoralism is the dominant production system. The total livestock population in the region, including resident herds and animals seasonally moving through the area, is estimated at 15–20 million heads [16]. Camels regularly move through and congregate at markets and slaughterhouses in Bol, creating opportunities for close contact among animals, ticks, and humans that may facilitate pathogen transmission. Despite the epidemiological importance of this setting, data on camel-associated ticks and selected tick-borne pathogens in the Lac region remain limited.

Therefore, this study aims to characterize the diversity of ticks and their associated tick-borne pathogens in Chadian camels, providing a basis for evidence-based control strategies.

## 2. Materials and Methods

### 2.1. Study Area

**Figure 1:**
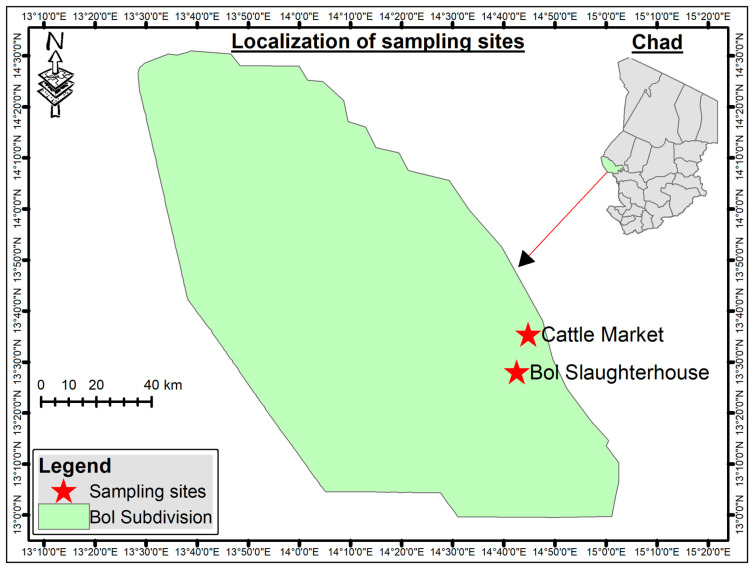
Location of the sampling sites in Bol, Lac region, Chad. Red stars indicate the animal market and the slaughterhouse where camels were examined for tick collection.

The study was conducted in the Lac region of Chad between July and August 2022. Sampling took place in the town of Bol at two locations: the animal market (13°28′078″ N, 14°42′495″ E) and the slaughterhouse (13°28′022″ N, 14°42′566″ E) (Figure 1). The Lac region has a Sahelian climate, with a long dry season and a short rainy season. Sampling was conducted during July–August, corresponding to the main rainy season in the Lake Chad basin, when environmental conditions are favorable for tick activity.

### 2.2. Tick Collection and Preservation

A total of 316 camels were examined, including 257 animals from the animal market and 59 from the slaughterhouse. The difference in numbers reflects animal availability during the sampling period, as substantially more camels were present and accessible at the market than at the slaughterhouse. Ticks were systematically collected using sterile fine-tipped forceps. Multiple body regions were inspected, including the ears, neck/dewlap, axilla, groin, perineal region, and tail base, to reduce collection bias toward a single attachment site. All collected ticks were adults; immature stages (larvae and nymphs) were not observed during field inspection. To standardize sampling effort, a maximum of five adult ticks per camel was collected. When ≤5 adult ticks were present, all were collected. When >5 was visible, ticks were collected from body sites in encounter order until the limit of 5 per camel was reached. Ticks were immediately preserved in individually labelled vials containing 70% ethanol. Prior to downstream analyses, specimens were rinsed with distilled water to remove residual ethanol and external contaminants. All tick collection procedures were conducted with institutional ethical approval (Application No. AN-STA-00001296).

### 2.3. Morphological Identification of Ticks

The morphological identification of tick species was based on key morphological characters, including scutal ornamentation, festoon coloration, groove patterns, leg banding, and the shape and position of adanal and subanal plates. Additional distinguishing features included setal density around the spiracle and the shape of the genital aperture. Ticks were examined following standardized taxonomic keys [17]. Only specimens with preserved diagnostic structures (“intact ticks”) were included for morphology-based species identification. Specimens were considered intact when the capitulum/mouthparts were undamaged and key characters (e.g., scutum, festoons, spiracular plates, adanal and subanal plates where applicable) were clearly visible; heavily damaged specimens were excluded from morphological assignment.

### 2.4. DNA Extraction

Ticks were initially washed with distilled water after removal from 70% ethanol to eliminate any debris and residual alcohol. Ticks were dissected into 4 equal pieces with a sterile scalpel blade, and DNA extraction was subsequently performed using the QIAGEN DNeasy Blood and Tissue Kit (Hilden, Germany) according to the manufacturer’s instructions. The quantity and quality of the extracted DNA were assessed using a Nanodrop spectrophotometer (Thermo Fisher,Waltham, MA, USA). Because tick tissues and residual blood may contain PCR inhibitors, therefore, silica-column extraction and only samples with good-quality DNA (A260/280 ratio 1.8–2.0 and A260/230 ≥ 2.0) were selected for downstream analysis. Extracted DNA was stored at −80 °C until further use.

### 2.5. Confirmation of Morphological Identification

A subset of 30 ticks was selected for molecular characterization based on the cytochrome c oxidase subunit 1 (*cox1*) gene. Specimens were chosen proportionally to species abundance: *Hyalomma dromedarii* (14/382), *H. rufipes* (7/176), *H. impeltatum* (6/149), and *H. truncatum* (3/73).

For molecular confirmation using PCR and sequencing, we followed the PCR conditions described by Chitimia et al. [18]. *Hyalomma truncatum* DNA (GenBank accession number PQ425563) [19] served as the positive control, while purified distilled water was used as the negative control. Specimens were chosen proportionally to species abundance.

### 2.6. Molecular Detection of TBPs

The 60 selected ticks were 30 from the animal market and 30 from the slaughterhouse. These 30 were randomly sampled in the respective group using Microsoft Excel’s random number generator. Samples were screened for SFG *Rickettsia* spp., *Coxiella burnetii, Anaplasmataceae*, and *Babesia/Theileria* spp. using primers targeting *ompB*, *IS1111*, *16S rRNA*, and *18S rRNA* genes, respectively. PCR conditions were optimized as previously described [20,21,22,23] (Table 1). DreamTaq PCR Master Mix (2X) (Thermo Fisher Scientific, MA, USA) was used for all PCR reactions. Sterile distilled water served as the negative control, while positive controls were *Anaplasma phagocytophilum*, *Rickettsia parkeri,* and a *Coxiella*-like endosymbiont of *Rhipicephalus microplus*, obtained from the Department of Entomology, University of Minnesota, Saint Paul, MN, USA.

### 2.7. Gel Electrophoresis and Sequencing

PCR products were analyzed on a 1.5% agarose gel stained with Ultrapure Ethidium Bromide (Invitrogen, Thermo Fisher, MA, USA) and visualized under UV light.

### 2.8. Amplicon Purification and Sequencing

Positive bands were excised using sterile scalpel blades, and DNA was purified using the Monarch Spin DNA Gel Extraction Kit (New England Biolabs, Ipswich, MA, USA). Purified DNA samples were sent to GENEWIZ (Azenta Life Sciences, South Plainfield, NJ, USA) for Sanger sequencing in both forward and reverse directions, using the same primers employed in the PCR reactions. Sequence editing and consensus sequence generation were performed using Geneious Prime version 2025.0 (Biomatters Ltd., Auckland, New Zealand). The resulting sequences were compared against the GenBank database using the BLASTn (V 2.17.10) program [24] available at http://blast.ncbi.nlm.nih.gov/Blast.cgi (accessed on 26 September 2025). A sequence identity of 98% or higher was considered the threshold for species identification of ticks and pathogens.

### 2.9. Phylogenetic Analysis

Multiple sequence alignments were conducted using MUSCLE in MEGA11 (version 11.0.13) with default parameters [25]. Phylogenetic analyses were performed using the maximum likelihood (ML) method implemented in IQ-TREE 2 (v2.3.6). The ModelFinder module of IQ-TREE 2 automatically selected the best-fit substitution model based on the Bayesian Information Criterion (BIC). ML analyses included 1000 ultrafast bootstrap replicates to assess branch support, ensuring robust phylogenetic inference. A phylogenetic tree was constructed for the *cox1* gene with *Amblyomma variegatum* (OK576094) as an outgroup. Final tree visualization and editing were performed using the Interactive Tree of Life (iTOL v7) [26].

### 2.10. Statistical Analysis

Using a binomial detection approach [27], the sample size required to detect at least one positive with 95% confidence when the true prevalence is ≥5% is n=ln(1−0.95)/ln(1−0.05)=58.4, rounded up to 59; therefore, screening 60 ticks provides approximately 95% confidence of detecting targets at ≥5% prevalence.

Associations between tick species distribution, sex ratios, and sampling locations (animal market vs. slaughterhouse) were analysed using chi-square tests of independence based on the full dataset of collected ticks (*n* = 780) in R (v4.3.2). For the molecular screening subset (n = 60), tick-borne pathogen prevalence was calculated as the proportion of PCR-positive ticks with 95% Clopper–Pearson confidence intervals. Pathogen results are presented descriptively for the screened subset.

## 3. Results

### 3.1. Tick Identification

A total of 316 camels were examined in Bol, Chad. Of these, 228 camels (72.2%) were infested with ticks, whereas 88 camels (27.8%) had no ticks detected. From the infested camels, a total of 780 ticks were collected, corresponding to a mean tick burden of 3.4 ticks per infested camel. These included 596 ticks (341 males, 255 females) at the animal market and 184 ticks (113 males, 71 females) at the slaughterhouse. Morphological identification classified the ticks into four *Hyalomma* species: *Hyalomma dromedarii* was the most abundant, representing 49.0% of all ticks (382/780; 213 males, 169 females), followed by *H. rufipes* (22.6%, 176/780; 102 males, 74 females), *H. impeltatum* (19.1%, 149/780; 93 males, 56 females), and *H. truncatum* (9.4%, 73/780; 46 males, 27 females) (Table 2).

**Figure 2:**
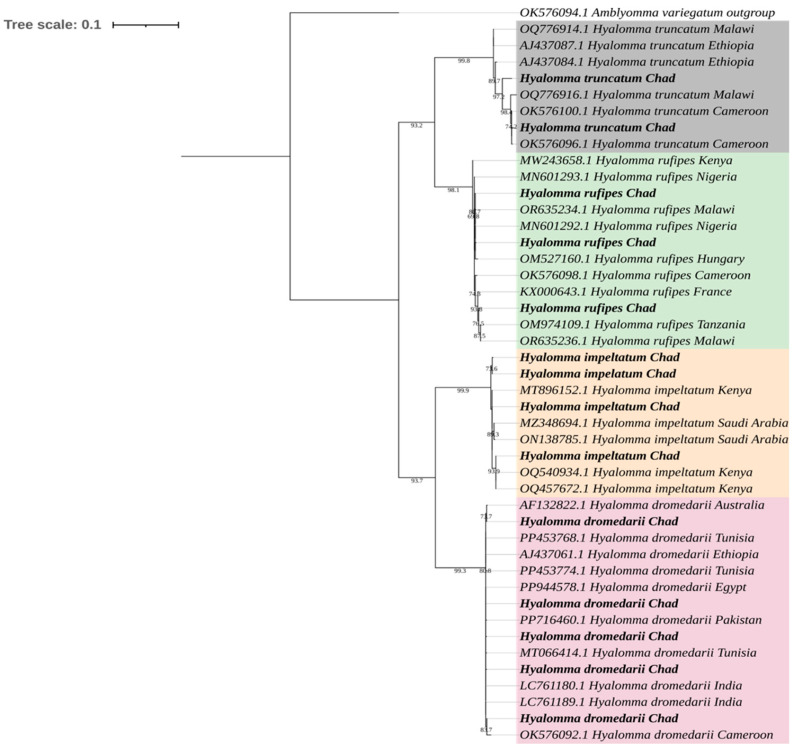
Maximum likelihood (ML) phylogenetic tree of the *Hyalomma dromedarii*, *Hyalomma impeltatum*, *Hyalomma rufipes* and *Hyalomma truncatum* tick species based on the *cox1* gene marker. Sequences obtained from this study are highlighted in bold, and different clades are distinguished using color highlights. *Amblyomma variegatum* (accession no. OK576094.1) was used as the outgroup.

Sequencing of *cox1* via PCR on the collected ticks confirmed all morphological identifications. The maximum likelihood tree revealed well-supported, species-specific clades (bootstrap > 70%) (Figure 2). All consensus sequences were submitted to GenBank, and their corresponding accession numbers are provided (PV770314-PV770324, PV696902-PV696908). Pathogen and endosymbiont distribution by tick species, location, and sex is detailed in Table 3.

### 3.2. Detection of Tick-Borne Pathogens

A total of 60 *Hyalomma* ticks were screened for selected tick-borne microorganisms. Eight ticks (13.3%; 95% Clopper–Pearson confidence interval [CI]: 5.9–24.6%) tested positive for at least one microorganism. *Coxiella burnetii* was detected in 7 ticks (11.7%; 95% CI: 4.8–22.6%), while *Rickettsia aeschlimannii* was identified in 1 tick (1.7%; 95% CI: 0.04–8.9%). The endosymbiont *Candidatus Midichloria mitochondrii* was detected in 6 out of 60 ticks (10.0%; 95% CI: 3.8–20.5%).

All screened ticks were negative for *Babesia* and *Theileria* (0/60; 95% CI 0–4.9%). The Anaplasmataceae-specific *16S rRNA* assay did not detect any pathogenic *Anaplasma* or *Ehrlichia* species (0/60; 95% CI 0–4.9%). The distribution of detected microorganisms across tick species, sex, and sampling location is presented in Table 3**.**

## 4. Discussion

We did not find significant differences in sex by species, except for *H. impeltatum*, which showed a significant male bias. This is likely because female ticks detach after feeding to lay eggs, while males detach briefly after feeding but frequently reattach to seek mates. This behaviour consequently increases the likelihood of males being detected on their hosts [28]. Although our sampling protocol (limited to five ticks per camel) could have favoured the collection of more mobile male ticks, this pattern aligns with observations in Kenya [29] and Nigeria [30], reflecting sex-specific differences in host attachment duration among *Hyalomma* species.

*Hyalomma dromedarii* was the dominant tick species, representing 49.0% of ticks collected, reinforcing its role as the primary tick parasite of dromedary camels [31,32]. This dominance is consistent across Africa, Nigeria [33,34,35], Kenya [29,30], and Egypt [36,37], as well as in the Middle East and Sudan [38]. Because camels congregate at markets and slaughterhouses, these sites can increase opportunities for tick transfer between animals, which may contribute to maintaining high tick burdens. Although camels are the preferred host, *H. dromedarii*’s ability to infest sheep, goats, cattle, and horses [17] highlights its ecological versatility and epidemiological significance in mixed livestock systems. *Hyalomma rufipes* is the second most prevalent species (22.6%), and similar results have been reported before in Nigeria (20.3%) [12] and Kenya (31%) [29]. However, regional and seasonal differences have been reported in Kenya [39]. This discrepancy may reflect ecological differences, as arid conditions in Marsabit and Samburu favor *H. rufipes*, which thrives better in dry habitats [17]. Our market context, involving camels from diverse regions, may dilute *H. rufipes* prevalence by introducing ticks from less arid areas. Abiotic factors like temperature, humidity, and host movement patterns also influence tick distribution [40,41], with seasonal sampling differences potentially explaining higher *H. rufipes* prevalence in dry seasons [1]. *Hyalomma impeltatum* ranked third (19.1%), consistent with its presence on camels across Africa and the Middle East, varying between 8.5 and 23% [9,29,39]. However, lower prevalences have been reported in Egypt (1.01%) [42], Nigeria (3.0%) [30], and Ethiopia’s Somali region (1.2%) [43]. Historically, a parasite of cattle and sheep [44,45], *H. impeltatum*’s presence on camels likely results from shared grazing and water sources in these regions. *Hyalomma truncatum* was least prevalent (9.4%), consistent with reports from Nigeria (14.67%) [30], Ethiopia (2.8%) [46], and Kenya (0.96%) [29]. Primarily a cattle tick [19,47,48], its prevalence on camels likely stems from mixed grazing. The low prevalence is attributed to competition from species that are adapted to camels.

All collected specimens were identified as *Hyalomma* spp., with no other tick genera detected. This finding aligns with numerous surveys focused on camels in arid and semi-arid regions, where *Hyalomma*, particularly *H. dromedarii*, is typically the dominant tick species found on camels. In these surveys, only *Hyalomma* spp. were identified [34,49,50]. However, other tick genera have occasionally been reported on camels, including *Rhipicephalus*, *Amblyomma*, and *Haemaphysalis* species, particularly in regions where camels share grazing areas with cattle and small ruminants [5,29,51]. In addition, our sampling occurred during the rainy season and focused on adult ticks collected from two high-throughput aggregation points (market and slaughterhouse), which may influence the observed tick fauna. Broader year-round sampling, including immature stages and a wider set of herds and habitats, may detect additional genera.

The importance of ticks also lies in their role in transmitting tick-borne pathogens. *Coxiella burnetii*, as identified by *IS1111* sequence analysis, was detected in *H. dromedarii* (3/60), *H. rufipes* (1/60), *H. impeltatum* (2/60), and *H. truncatum* (1/60) at an overall camel tick prevalence of 11.7% (95% CI: 4.8–22.6). This matches a relatively recent meta-analysis of a prevalence of 2.91–13.97% in African ticks and 4.76–12.53% in Middle East ticks [52]. In contrast, some recent studies on camel-associated *Hyalomma* ticks in Egypt, Kenya, and the UAE have reported a slightly higher prevalence [15,36,53]. Although *C. burnetii* has been detected in camel-associated ticks, human infection is thought to occur mainly through inhalation of contaminated aerosols and exposure to infected animal secretions rather than through tick bites [53,54]. Reviews suggest that ticks may contribute to the maintenance of *C. burnetii* in natural cycles involving wildlife and livestock; however, their epidemiological importance in Q fever transmission appears limited, and several studies have reported no detection of the pathogen in sampled tick populations [55,56].

*Rickettsia aeschlimannii,* a spotted fever group rickettsia, causing Mediterranean spotted fever-like illness, was detected in one *H. rufipes* tick (1/60, 1.7%, 95% CI: 0.04–8.9) from a market. This low prevalence aligns with a prior study in Chad [57], but contrasts with higher rates in Algeria (38.4%) [58], Nigeria (36.8%) [30], and Senegal (44.8–51.3%) [59]. *R. aeschlimannii* has also been detected in *H. dromedarii* in Tunisia (50%) [60], Nigeria (6.2%) [30], and Israel (2.7%) [61]. The low prevalence may reflect seasonal effects on tick survival and host availability [48], as well as the limited sample size and single-season sampling design. In addition, because ticks were collected from multiple camels and represented four *Hyalomma* species, the effective sample size for species-specific inference was smaller than the total number screened; therefore, the species-level distribution of detected microorganisms should be interpreted as descriptive rather than definitive. Because camel tick burdens can vary substantially across months and seasons, our sampling should be viewed as a seasonal snapshot rather than a year-round estimate.

No *Babesia* spp. or *Theileria* spp. were found in *Hyalomma* ticks, consistent with results from Kenya and Saudi Arabia [9,29,62]. Their prevalence in ticks has always been low [30,63]. However, the relatively small screened subset (n = 60) and the cross-sectional nature of the sampling limit the ability to detect pathogens with low prevalence.

We also found *Candidatus Midichloria mitochondrii,* through sequence analysis of the *Anaplasmataceae* *16S rRNA*. This bacterium is an endosymbiotic of ticks. This unculturable bacterium lives in the mitochondria of ticks and has been reported from various countries [64,65,66,67] and from various tick species [68,69,70,71]. *Candidatus Midichloria mitochondrii* DNA has also been detected in 34.1% of tick-exposed individuals in Italy without clinical symptoms [72]. Its detection here is therefore interpreted as part of the tick microbiome rather than evidence of a tick-borne pathogen.

## 5. Conclusions

This study provides baseline data on camel-associated *Hyalomma* ticks in Bol (Lac region, Chad), confirming the predominance of *H. dromedarii* and the presence of three additional *Hyalomma* species. Screening of a subset of ticks detected *Coxiella burnetii* and *Rickettsia aeschlimannii*, while no piroplasms or pathogenic Anaplasmataceae were identified. These findings contribute to understanding tick composition and selected tick-borne pathogens in an important livestock aggregation zone. Future studies incorporating larger sample sizes and multi-season sampling will help refine prevalence estimates and clarify epidemiological dynamics in transhumant camel systems. The results support continued surveillance and integrated tick control strategies within a One Health framework.

## Figures and Tables

**Table 1:** Primer Details for Molecular Identification of Tick Species and Tick-Borne Pathogens.

Identification Target	Primer Name	Target Gene	Sequence (5′-3′)	Approximate Size (bp)	Reference
Tick species	Cox1F Cox1R	*cox1* gene	GGAACAATATATTTAATTTTTGG ATCTATCCCTACTGTAAATATATG	820	[18]
*Rickettsia* spp.	*120-2788* *120-3599*	*Rickettsia ompB*	AAACAATAATCAAGGTACTGT TACTTCCGGTTACAGCAAAGT	856	[23]
*Coxiella burnetii*	*Trans1* *Trans2*	*Coxiella IS1111*	TGGTATTCTTGCCGATGAC GATCGTAACTGCTTAATAAACCG	687	[21]
*Anaplasmataceae*	*EHR16SD* *EHR16SR*	*16S rRNA*	GGTACCYACAGAAGAAGTCC TAGCACTCATCGTTTACAGC	345	[22]
*Babesia* spp./*Theileria* spp.	*RLB F* *RLB R*	*18S rRNA*	GAGGTAGTGACAAGAAATAACAATA TCTTCGATCCCCTAACTTTC	460	[20]

**Table 2:** Distribution of Tick Species by Location and Sex.

Species	Location	Male	Female	Total per Location
*H. dromedarii*	Animal Market	154	128	282
	Slaughterhouse	59	41	100
Total		213	169	382 (49.0%)
*H. rufipes*	Animal Market	79	60	139
	Slaughterhouse	23	14	37
Total		102	74	176 (22.6%)
*H. impeltatum*	Animal Market	73	44	117
	Slaughterhouse	20	12	32
Total		93	56	149 (19.1%)
*H. truncatum*	Animal Market	36	22	58
	Slaughterhouse	9	6	15
Total		46	27	73 (9.4%)
All Species	Animal Market	341	255	596
	Slaughterhouse	113	71	184
Grand Total		454	326	780

Overall sex ratio was (454 males to 326 females, with no significant differences by location (*p* = 0.534) or species (*p* = 0.385).

**Table 3:** Prevalence of Tick-Borne Pathogens and Endosymbiont in 60 Ticks by Species, Sex, and Location.

Pathogen/Endosymbiont	Total	*H. dromedarii*	*H. rufipes*	*H. impeltatum*	*H. truncatum*	Total Positive	Location (Market/ Slaughterhouse)
*Coxiella burnetii*	60	3 (2M/1F)	1 (0M/1F)	2 (1M/1F)	1 (0M/1F)	7 (11.7%) [4.8–22.6]	5/2
*Rickettsia aeschlimannii*	60	0	1 (0M/1F)	0	0	1 (1.7%) [0.04–8.9]	1/0
*Candidatus Midichloria mitochondrii* (Endosymbiont)	60	3 (1M/2F)	1 (1M)	2 (1M/1F)	0	6 (10.0%) [3.8–20.5]	4/2
Total Positive Ticks	60	6	3	4	1	14 (23.3%) [5.9–24.6]	10/4

Note: M = Male, F = Female. Percentages calculated as positives per species tested, with 95% Clopper-Pearson CIs. Location shows positives per pathogen (animal market/slaughterhouse).

## Data Availability

The original contributions presented in this study are included in the article. Further inquiries can be directed to the corresponding author.
